# Ill Effects of a Small Aneurism of the Aortic Arch Illustrated

**Published:** 1887-03

**Authors:** Henry F. A. Goodridge

**Affiliations:** Senior Physician to the Bath Royal United Hospital


### Clinical Records.

---

### ILL EFFECTS OF A SMALL ANEURISM OF THE AORTIC ARCH ILLUSTRATED. By HENRY F. A. GOODRIDGE, M.D., F.R.C.P., Senior Physician to the Bath Royal United Hospital.

J. D., æt. 34, was admitted into the Bath Royal United Hospital on November 25th, 1884. Until two years previously he was in the Indian Army. Since then he had been a stonemason, or rather a dresser of free-stone, and for the same period he had suffered more or less from cough. Eight months prior to admission, being at that time employed in Paris, his cough and failure of health were such that he was taken into the British Hospital there, and remained ten weeks. He got better in the ensuing summer, but not quite well. For now, however, nearly two months he had become decidedly worse. His cough, which had much increased, had been attended with the expectoration of a large quantity of more or less opaque sputa, but with no hæmoptysis; he had sweated a good deal at night; he had latterly lost much flesh; for nearly a week his appetite had failed; and the last few nights he had had no sleep. His bowels had been regular. He continued his work until three weeks, and did not keep his bed until one week, before admission. His state at this date was as follows: He was suffering from considerable dyspnœa without any stridor, and he had a frequent, thick, suffocative cough; his voice was weak, but not otherwise remarkable. He had some herpetic eruption on his lower lip; his pupils were normal; there was no distension of the cervical veins. His temperature in the axilla was 98·4°; his pulse was 112; his respirations were 28. On examination of the chest, hyper-resonance on percussion was observed at both bases posteriorly and about the spine of the left scapula; also at the sternal notch, and more or less over the left front; varying moist and dry rhonchi were heard behind with inspiration, particularly towards the bases, the moist sounds being all fine; the inspiration was very rhonchal under both clavicles—it cleared a little on coughing under the left, but not under the right; the expiration was very prolonged in the latter situation. The cardiac dulness was much obliterated, and the heart's apex-beat was not distinct either to sight or to the feel; the sounds at the apex had rather a tic-tac character; no murmur was audible anywhere. The hepatic dulness reached half-way to the umbilicus. His bowels were now somewhat sluggish; his urine was acid, of sp. gr. 1.027, and showed a trace of albumen. His feet were cold, but were free from œdema. He was ordered to be dry-cupped over the chest, and to take zinci sulph. gr. xx. ex aquâ calidâ the same evening, and pulv. rhei sal. ʒi. the following morning. 8.30 p.m.: He had vomited freely after the emetic, but he had scarcely less dyspnœa. His temperature now was 102°.

Nov. 26th.—He had slept very little, had sweated a good deal, and had still much dyspnœa, with an abortive suffocative cough. His temperature was 100° (evening, 100·6°). His tongue was coated with white fur, and he was taking food badly. He was ordered acidi nitrici dil. ♏ xx. + extr. cinch. flav. liq. ♏ xv. + acidi hydrocy. dil. ♏ iv. + syr. scillæ ʒss. ex aquæ ℥i. every six hours, and to have six ounces of wine added to his diet.

27th.—He had slept altogether about one hour. After sleeping for a few minutes only, he awoke nearly suffocated, and sweating profusely. He was expectorating thick puriform masses. He took his milk, wine, and other fluids fairly, but no solid food. He was ordered chloral hydr. gr. x. ex aq. camph. at bed-time, and to be repeated, if necessary, every second hour for three doses.

28th.—He had dozed a good deal the preceding day, but had slept during the night only one hour and a half altogether. His cough was much the same, and attended with the expectoration of copious greenish nummular sputa. Subcrepitant rhonchus was heard under the right clavicle; under the left was less rhonchus, but the breathing there was harsh and exaggerated; sonorous rhonchus was present all about the left base. His urine was of sp. gr. 1.030; it deposited lithates, but contained no albumen. He was ordered to take ammon. carb. gr. xxx. ex aquâ calidâ the same evening.

30th.—As the carbonate of ammonia on the 28th had no emetic effect, he took the previous evening zinci sulph. gr. xx., and vomited freely, but was much oppressed in his breathing for an hour afterwards. He had slept about two hours. He seemed now rather easier; his cough was hardly so troublesome. His temperature was 99·8°, his pulse was 108, and his respirations were 32.

December 1st.—He had slept two hours; his breathing was still difficult; his cough was rather less; the sputa were of the same character, but less in quantity. His breathing was more free from rhonchus. His bowels were not open. He was ordered to take pil. hydrarg. gr. iii. + pil. colocynth. et hyoscy. gr. vii., made into two pills, at 4 p.m.

2nd.—He had not slept at all, and seemed worse. His cough was almost incessant, and his dyspnœa was most distressing. His temperature was 99°. At the visit his complexion was noticed to be rather dusky; his pulse was 140; there were large mucous and submucous rhonchi at both bases, but the inspiratory sound was rather better in front. He was taking his liquid food well; his bowels had been opened twice; his urine was acid, of sp. gr. 1.035, and was free from albumen. At 6 p.m. he was sweating profusely; had most urgent dyspnœa—was, indeed, gasping for breath; his face was livid. He was ordered to have immediately liq. strychniæ ♏ iii. injected hypodermically, to discontinue the draught, and to have instead the following every four hours: ℞. liq. strychniæ ♏ iv., ammon. carb. gr. v., tinct. scillæ ♏ xx., aq. camph. ad ℥i. ♏. ft. haust.

3rd.—He had not slept more than one hour, and had been delirious more or less through the night, and was still so. His dyspnœa, however, was now distinctly relieved, and his cough and expectoration were less; he was also less livid in the face. His temperature was 100.4°; his pulse was 132; his respirations were 32.

4th.—He had slept about five hours continuously in the early part of the night; he was then awakened for food, and had only dozed a little since; he had had no delirium. His cough was less troublesome, though he had a bad bout of it just after waking; he had only slight expectoration. His temperature was 99° (evening, 100°); his pulse was 120; his respirations were 36. He was taking his food well.

5th.—He had slept only about one hour and a-half; he had had no delirium. He had coughed rather more, and had expectorated a little more frothy muco-purulent sputa. He was still dusky in the face. His temperature was 100°; his pulse 144; and his respirations 36. There was large bubbling rhonchus audible under both clavicles. 8 p.m.: His breathing was very laboured; his pulse was 132. He died at midnight.

On *post-mortem* inspection, the lungs exhibited evidences of intense bronchitis, with consolidation of the bases by catarrhal pneumonia. An aneurism, the size of a walnut, was found projecting from the posterior surface of the transverse part of the arch of the aorta. It had a round well-defined orifice, rather less than an inch in diameter, and no coagulation had taken place in the sac. The aneurism did not involve the origin of the vessels, being below the level of the same; but in its situation vertically it corresponded to the interval between the innominate and left carotid arteries. It pressed directly backwards on the trachea just above its bifurcation, and had the left recurrent laryngeal nerve quite near it, without, however, being compressed by it. Patches of atheroma were found in the aorta. The heart was healthy, as were also the kidneys, liver, and spleen. A little pigmented spot was present on the glans penis, otherwise there were no signs of syphilis.*

We remark first in the foregoing case that the patient was the subject of bronchitis. The origin of it seems to have been connected with his occupation of dressing free-

* The trachea and the aortic arch with the aneurismal protrusion, which were removed together in undisturbed apposition, I had myself the opportunity of inspecting; but for the other necroscopic particulars, as well as for much of the clinical record, I am indebted to our able house-physician, Dr. Edward Cave.—H. F. A. G.

stone, a dusty operation, in which the workman is liable to inhale the irritating particles of stone set free, and to suffer therefrom, as we in Bath have special opportunities of observing. He had not so very long been thus employed, not long enough to bring about those characteristic structural changes of lung-tissue which we know ultimately supervene in these and such-like cases, but sufficiently long to set up a chronic bronchitis with attendant emphysema. Exacerbations occurred from time to time, and his general health and nutrition had become considerably impaired. It was in one of these exacerbations, as it then seemed, that he was admitted into the Bath Hospital: it was undoubtedly a severe one, the fine capillary bronchioles being especially involved. Yet there appeared at first to be no reason why relief should not ensue from appropriate treatment. As was ascertained after death, however, and as was at length surmised during life, the patient's malady was not uncomplicated. He had a small (size of walnut) sacculated aneurism of the arch of the aorta, which pressed on the trachea. It was too small to compress or narrow the tube, and cause stridor; too small, perhaps, to furnish any direct sign or symptom whereby its presence could with any certitude be revealed; but it was not too small to interfere by its pressure with the efficient performance of expectoration, upon which recovery from the severe bronchial catarrh mainly depended. There was a hitch, an impediment, in the act of expectoration, very manifest latterly on attentive observation of the patient, and it was clearly situated high up in the air-passages. Emetics, usually so beneficial in capillary bronchitis, were of but little avail; strychnia, on the contrary, by goading, probably, for a short interval the bronchial muscle to unwonted exertion, afforded marked, though transient, relief. The patient's cough was abortive, and the products of inflammation of the bronchial mucous membrane were inadequately expelled. Hence ensued accumulation in the tubes, with its asphyxiating tendency; while the same products inhaled into the pulmonary alveoli doubtless set up inflammatory changes therein, if, indeed, these had not already been initiated by extension from the bronchioles themselves. Thus lobular, catarrhal, or broncho-pneumonia supervened, and death followed. We know that this is one of the ways in which larger aneurisms in the same situation now and then produce their fatal effects.

The case teaches, I think, how important in the mechanism of expectoration is the spot where the confluence of the expiratory currents from the two lungs takes place; and that the free and unrestricted play of the trachea at that spot is as essential to the efficiency of expectoration as are ciliary action, pulmonary elasticity, and contractile energy of respiratory muscles, in due amount respectively, on the one hand, and proper closure of the glottis on the other.

I would add that many years ago I had a somewhat similar case, where was latent, not an aneurismal tumour, but an enlargement of bronchial glands; and its recollection led me at length to suspect the existence of something of the kind in the present instance. The same peculiar difficulty of expectoration with little or no relief from emetics, in a case of capillary bronchitis or "suffocative catarrh," as it is sometimes called, would certainly lead me in the future to diagnose a similar complication.

